# Factors Likely to Affect the Uptake of Genomic Approaches to Cancer Screening in Primary Care: A Scoping Review

**DOI:** 10.3390/jpm12122044

**Published:** 2022-12-10

**Authors:** Kaitlyn V. Davis, Mie H. Hallman, Melissa DiCarlo, Sophie M. Wambua, Rachel L. Jaffe, Allison W. Welsh, Cameron Kerber, Hushan Yang, Christopher V. Chambers, Ronald E. Myers

**Affiliations:** 1 Department of Family and Community Medicine, Thomas Jefferson University, 1015 Walnut St., Philadelphia, PA 19107, USA; 2 Department of Medical Oncology, Thomas Jefferson University, 834 Chestnut St., The Franklin Building, Suite 314, Philadelphia, PA 19107, USA; 3 Exact Sciences Corporation, 5505 Endeavor Lane, Madison, WI 53719, USA

**Keywords:** liquid biopsy, multi-cancer early detection, primary care, early detection of cancer, cancer screening tests

## Abstract

Genomic tests are being developed for use in cancer screening. As most screening is offered in primary care settings, primary care provider and patient perceptions of such tests are likely to affect uptake. We conducted a scoping review to synthesize information on factors likely to affect patient and provider use of biospecimen collection and analysis for cancer screening, methods referred to as liquid biopsy or multi-cancer early detection (MCED) testing when used to detect multiple cancers. We ultimately identified 7 articles for review and analyzed them for major themes. None reported on primary care provider perspectives. Six articles focused on patient perceptions about testing for a single cancer (colorectal), and 1 reported on patient views related to testing for multiple cancers. Factors favoring this type of testing included its non-invasiveness, and the perceived safety, convenience, and effectiveness of testing. There is a dearth of information in the literature on primary care provider perceptions about liquid biopsy and MCED testing. The limited information on patient perceptions suggests that they are receptive to such tests. Research on primary care provider and patient test-related knowledge, attitudes, and behavior is needed to guide future implementation in primary care settings.

## 1. Introduction

Cancer is the leading cause of death worldwide and the second leading cause of death in the United States. In 2022, there will be an estimated 1,918,030 cancer cases diagnosed and approximately 609,360 deaths from cancer in the country [1,2]. Among cancers for which screening tests are currently recommended [3,4,5,6], the distribution of early stage disease at diagnosis is as follows: female breast cancer (65%), uterine cervix (44%), colorectal (34%), and lung (24%) [1]. The development of new minimally invasive methods for testing biospecimens (e.g., blood, saliva, urine, stool samples) for genomic characteristics may provide additional tools to screen for these and other types of cancer [7,8].

Lokshin et al. [9] explain that such biomarkers can identify changes in the “genetic, epigenetic, proteomic, glycomic, and metabolomic profile of normal tissues”. When performed using biofluids to identify cancer-specific biomarkers, such testing has been referred to as “liquid biopsy” [10]. These tests have the potential to detect one type of cancer or to detect multiple cancer types [10]. When used to detect multiple cancers, the tests are often referred to as “multi-cancer early detection” (MCED) tests [11]. It is hoped that liquid biopsy and MCED tests can help to find early stage, curable cancers for which there are currently recommended screening tests (i.e., breast cancer, cervical cancer, colorectal cancer, and lung cancer) and for which there are no currently recommended screening tests (e.g., ovarian cancer and pancreatic cancer). It is not yet known, however, if the widespread use of such tests will reduce cancer morbidity and mortality [12,13]. 

Recent reviews have highlighted factors that are likely to influence the uptake of currently recommended cancer screening tests, including provider support of testing and patient access to and views of screening [14,15]. Little is known, however, about factors that are likely to affect primary care provider and patient uptake of new genomic approaches for cancer screening. We conducted a scoping review to learn what has been reported in the literature on those factors.

## 2. Materials and Methods

Our research team based the scoping review on the following question: “What factors are likely to affect patient and provider decisions for the use of liquid biopsy testing in cancer screening?”. Initially, two members of our team (KVD and RLJ) worked with a health sciences librarian at Thomas Jefferson University to develop a search strategy that focused on publications appearing in the literature from January 2000 through June 2021.

We used the Boolean operator OR to link terms in an initial group (i.e., patient, provider, primary care) and those in a second group (i.e., liquid biopsy, multi-cancer early detection or MCED, and multi-analyte testing). We did not include terms that described the nature of biospecimen collection for testing. The research team also used the Boolean operator AND to link the first group to the second group. Further discussion led the research team to modify the search by refining the scoping review question to: “What factors are likely to affect primary care provider and patient use of liquid biopsy testing to detect a single, defined cancer or for multiple cancers, and provider and patient use of MCED testing?”.

**Figure 1:**
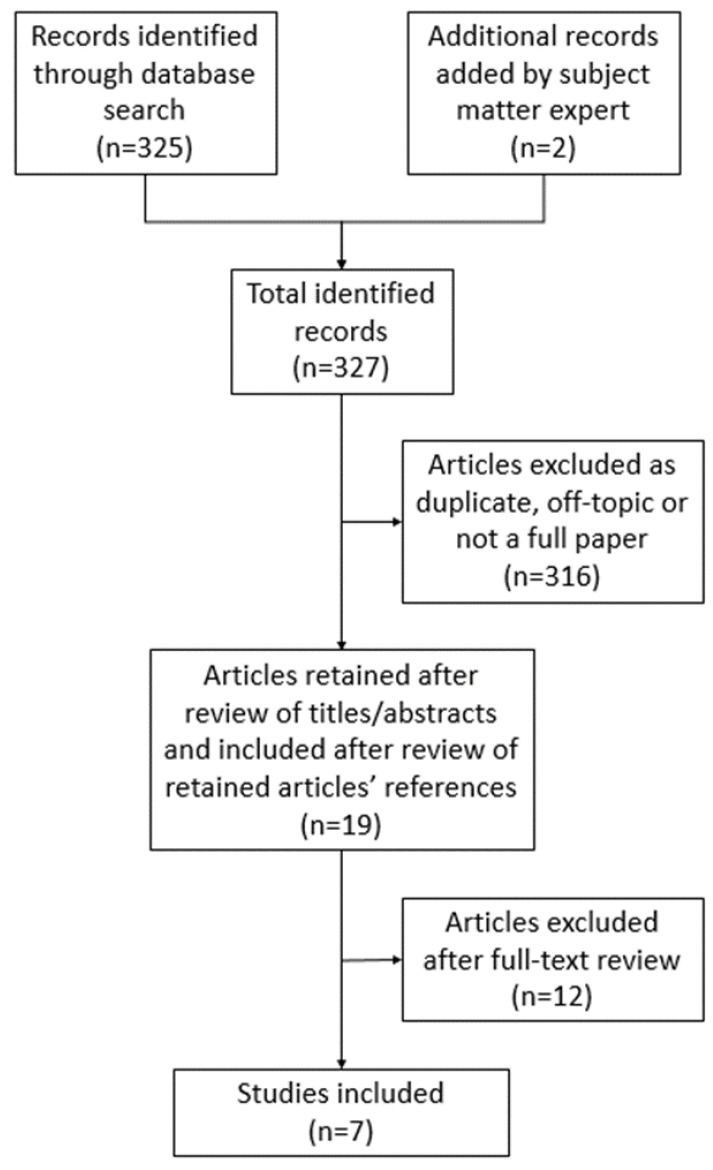
Scoping review schema. This represents each step taken to complete the scoping review. The schema is based on the Preferred Reporting Items for Systematic Reviews and Meta-Analyses Extension for Scoping Reviews (PRISMA-ScR).

The final search focused on electronic databases that included PubMed, Ovid, and Scopus, and used the following terms: “multi-cancer screening”, “liquid biopsy”, “multi-cancer early detection”, “MCED”, and “multi-analyte testing”. We used the Preferred Reporting Items for Systematic Reviews and Meta-Analyses Extension for Scoping Reviews (PRISMA-ScR) published by Tricco et al. [16] to guide the review strategy. We utilized their PRISMA-ScR Checklist to record each step of our scoping review as follows (see Figure 1):

1. The research team performed an initial search on the databases PubMed, Ovid, and Scopus, followed by the manual removal of duplicates across the results. Additionally, a subject matter expert on the research team (REM) suggested the inclusion of 2 additional articles. Search terms included: “Multi-cancer screening” or “Multi-cancer early detection” or “MCED” or “multi-analyte blood testing” or “multi-analyte blood test” or “multi-analyte assay”.
2. Members of the team (KVD and MHH) reviewed the search results based on title and abstract. Articles that were not related in any way to the perception of liquid biopsy or MCED were excluded.
3. Members of the team (KVD and MHH) reviewed the full text of each of the remaining articles. Any article that did not discuss patient or provider views related to the use of liquid biopsy or MCED testing were excluded.
4. Members of the team (KVD and MHH) reviewed references of the included articles for any other relevant articles.
5. Members of the team (KVD, MHH, and REM) analyzed the final articles, recorded details of each study, and summarized key themes (see Table 1).

## 3. Results

The initial search across the 3 databases, including the 2 suggested articles, returned 327 articles. We removed 117 duplicates for a total of 210 articles. We then excluded 200 articles after a review of their titles and abstracts. We reviewed the full text of the remaining 10 and excluded 7 articles. We reviewed the references of the 3 remaining publications to identify any other relevant articles [17,18,23]. As a result, we added 4 more publications [19,20,21,22]. Thus, the scoping review resulted in the identification of 7 articles for analysis and synthesis.

### 3.1. Provider Perspective

We found no articles in the literature that reported on primary care provider perspectives related to liquid biopsy or MCED test use in cancer screening.

### 3.2. Patient Perspective

The scoping review resulted in the identification of 7 articles that reported patient perceptions about liquid biopsy or MCED test use in cancer screening [17,18,19,20,21,22,23]. The articles included patients who tended to be 50 to 80 years of age, a segment of the population at increased risk for cancer and eligible for screening. Six of the articles focused on single cancer (colorectal cancer) screening. The studies reported survey data (N = 6) and observational data (N = 1) related to patient perspectives on liquid biopsy and MCED testing.

Of the 7 articles included in the review, 6 compared patient views of liquid biopsy screening for a single cancer to other cancer screening options [17,18,19,20,21,22]. Here, liquid biopsy methods included either providing a blood sample or completing a standard stool blood test or sDNA stool blood test, both of which were intended to detect only one type of cancer. Five of the articles reported on studies in which patients responded to a survey comparing liquid biopsy to current cancer screening modalities used in colorectal cancer screening [17,19,20,21,22]. The other article reported on results of an observational study in which the uptake of either a stool test or blood test for colorectal cancer was compared among patients who refused a colonoscopy [18]. These articles reported that patients were receptive to newer biospecimen screening methods because of familiarity with biospecimen collection procedures, relative comfort, convenience, and the minimally invasive nature of the test. It was also reported, however, that patients preferred colonoscopy over liquid biopsy for colorectal cancer screening, because they viewed colonoscopy as the more accurate screening test [21]. 

One article reported survey results on patient receptivity to the use of a multi-organ DNA blood test for cancer screening [23]. The survey asked participants if they would use a multi-organ stool DNA blood test to detect gastrointestinal cancers and solicited reasons for their response. Almost all (98%) participants reported that they would use this type of test over standard of care screening tests [23]. The most frequently cited reasons were the perceived capacity of the new test to detect multiple cancers, as well as the convenience and safety of this type of testing [23].

### 3.3. Perceptions of Liquid Biopsy and MCED Testing in Diverse Populations

One of the articles provided information on the perceptions of white and African American participants who responded to a survey on genomic cancer screening. In this study, Abola et al. [17] asked survey respondents about their preference for sDNA, fecal occult blood test (FOBT), or colonoscopy screening for colorectal cancer. Respondents indicated if they preferred one of the tests, if they had no preference, or if they were unsure. No statistically significant differences by race were found after controlling for sociodemographic factors [17]. In another study, Schroy and Heeren [21] reported that patients had a positive perception of sDNA testing, and that this view was comparable across racial groups.

## 4. Discussion

We conducted this scoping review to assess what has been reported in the literature on patient and provider perceptions related to liquid biopsy and MCED test use in cancer screening. At the time of the review, we determined that there were no published studies that reported on primary care provider views of this approach to early cancer detection. The lack of research on provider perceptions in this area is not surprising, given that liquid biopsy and MCED test development for cancer screening is a relatively recent phenomenon. This gap in knowledge is important; given the likelihood that such testing will become available for use in concert with standard of care screening in the not too distant future [24]. The use of liquid biopsy and MCED tests that have high sensitivity and specificity, along with screening tests currently offered in primary care, has the potential to be highly effective, and pressure for their use is mounting.

Some studies have identified potential barriers to the incorporation of genetic testing in primary care. Such reports have identified a number of factors, including limited provider knowledge about genetic testing, the logistics of integrating genetic testing into the clinical workflow, the time and effort required to provide genetic testing, provider confidence in managing genetic test results, and health system-level issues [25,26,27]. A recently published study [28], not included in this scoping review, suggested that primary care providers may be especially receptive to liquid biopsy and MCED testing for cancer screening for use with patients who are unable to undergo invasive standard of care screening procedures, are concerned about the convenience of screening, and are reticent about undergoing standard of care screening. 

To date, the United States Food and Drug Administration (FDA) has granted approval for only one blood-based liquid biopsy test (Septin9) as a colorectal cancer screening test for use in concert with recommended screening tests. Importantly, there are no published guidelines on the use of genomic testing for single or multiple cancers in clinical practice. It is important to mention that the FDA has granted a number of genomic tests “breakthrough device” status, a designation that encourages expedited test development and evaluation. Current cancer screening guidelines do not address the use of such testing as part of standard of care screening. There is a pressing need for research on factors that are likely to affect provider uptake of the new genomic methods of cancer screening prior to widespread promotion of their use in the primary care landscape.

Findings from our review of studies that addressed patient perceptions highlight patient receptivity to liquid biopsy screening and MCED testing. These studies suggest that patients are likely to be receptive to these new approaches to cancer screening, especially if they are easy to do, convenient, and non-invasive; are as effective as currently recommended screening; and are recommended by a provider. These published results also suggest that patients value screening tests that can find more than one type of cancer. 

These observations are consistent with those reported in a recent study, which reported on survey results for 1700 patients 50 to 80 years of age who were recruited through a recruiter database, online panels, and social media [29]. Interestingly, that report noted that survey respondents also indicated a preference for MCED tests that were able to identify cancer at specific sites. In practice, it is likely that factors identified in our scoping review and in more recent publications, along with other factors, such as costs associated with testing and the nature of diagnostic follow-up, will affect patient uptake of genomic cancer screening tests.

Furthermore, it is possible that the uptake of liquid biopsy and MCED testing for cancer screening may vary among patients from different sociodemographic backgrounds. In this regard, we found only two studies that reported on opinions about such testingacross diverse patient populations. Importantly, the studies indicated that there were no racial differences in perceptions about liquid biopsy. Given the need to ensure equity in cancer screening, it is important to explore those factors that are likely to affect uptake of testing in primary care with patients from diverse populations more thoroughly. 

Regarding the introduction of liquid biopsy and MCED cancer screening in clinical practice, Ignatiadis et al. [30] pointed out that there are substantial challenges to be addressed. These challenges include the need to minimize the number of false positive and false negative test results, and address the emotional and financial effects on patients of receiving test results that indicate the presence of cancer and the subsequent need for diagnostic follow-up to confirm the finding. As noted by Etzioni, Gulati, and Weiss [31], it is also important to determine the effects of such testing in terms of over-diagnosis, as some cancers that are indolent and not life threatening may be detected. Moreover, there is much to learn about the impact of test use on mortality and survival related to specific cancers. Furthermore, it is important to identify effective strategies to address obstacles to implementation in practice. A collaborative effort involving the public and private sectors, along with health systems, providers, and patients is needed if the promise of genomic test use in cancer screening is to be realized. 

We limited studies included in this review to those related to primary care providers and patients and that focused on cancer screening. We did not include other health care providers, such as specialists who use liquid biopsy tests in the course of treating patients who have been diagnosed with cancer to guide treatment decision making. In addition, we excluded reports that may have provided insights into perceptions of diagnosed patients regarding the use of such testing modalities. This decision was influenced by the view that perceptions of primary care providers and asymptomatic patients related to the detection of cancer are likely to differ from those of specialists and diagnosed patients who are actively engaged in treatment.

Furthermore, we excluded studies that may have reported on direct-to-consumer (DTC) genomic testing. We based this decision on the fact that such tools were not developed for the specific purpose of cancer screening, and, to our knowledge, are not being used in clinical care. It is also important to mention that this review generated little information about factors that might affect the use of liquid biopsy and MCED testing in health systems, among primary care practice settings, and with diverse patient populations.

A potential limitation of this report is that we focused the review on publications that appeared in the literature from 2000 to 2021. It is possible that other articles on the topic were published earlier than 2000. However, we found a small number of articles published during the search period and found no relevant articles published between 2000 and 2005. Given the recent emergence of liquid biopsy and MCED testing related to cancer screening, we believe that the publication of informative articles prior to 2000 is unlikely.

Cancer mortality rates could be reduced substantially by increasing the proportion of persons who are diagnosed with and treated for early stage neoplasia. Existing preventive health guidelines encourage screening for and treating early stage breast, cervical, colorectal, and lung cancer. As noted earlier, research is underway to develop and evaluate genomic approaches that can be used to identify persons with early stage cancer. It is hoped that, ultimately, research in this area along with studies on interventions to facilitate uptake in clinical practice will lead to substantial improvement in cancer prevention and control.

## Figures and Tables

**Table 1:** Scoping review article highlights. This table includes key findings from articles retained from the scoping review.

Author (Year)	Population and Sample Size	Study Design	Study Description	Outcomes
Abola et al., (2015) [17]	Patients 30–80 years of age at average risk for colorectal cancer (CRC) (n = 423)	Cross-sectional survey	Patients underwent both colonoscopy and stool DNA (sDNA) test, then completed a survey about their experience and preferences	75% of patients found sDNA test more suitable than a colonoscopy. sDNA testing was the preferred method among Caucasians (43%) and African Americans (32%) in univariate analysis. No racial differences in multivariate analyses related to preference. Reasons cited for sDNA preference included lack of bowel preparation, convenience, and ease of completion.
Adler et al., (2014) [18]	Patients 50–75 years of age at average risk for CRC (n = 172)	Observational study	Patients who refused colonoscopy were offered either stool test or epi proColon blood test	63% of patients refused colonoscopy. Of those, 15% chose having a stool test, 83% chose having an epi proColon blood test, and 3% refused both options. Top three reasons for refusing colonoscopy were: being uncomfortable with bowel preparation (54%), fear of colorectal cancer (44%), and fear that colonoscopy would be painful (32%). When asked why they favored one of the alternative screening tests, 78% and 81% of patients who had a blood test and stool test, respectively, said ease of getting tested influenced their choice.
Benning et al., (2014) [19]	Dutch adults 55–75 years of age (n = 815)	Case study using a discrete choice experiment (DCE)	Participants took an online survey where they were presented with attributes for blood, stool, or combination tests for colorectal cancer screening and asked to pick their preferred test	Test attributes that most affected a patient’s preference for it were: test sensitivity, supporting scientific evidence, and ability to reduce CRC death. A hypothetical molecular marker blood test combined with a standard stool test for colorectal cancer was preferred over the blood test or stool test alone. When comparing just the single test types, the hypothetical blood test was preferred over the stool test.
Berger et al., (2006) [20]	Patients whose doctors ordered an sDNA kit between August 2003 and July 2005 (n = 1211)	Cross-sectional survey	Patients answered a survey about their experience with the sDNA test	A majority of patients found the collection (64%) and return (74%) of the sample to be very easy. A majority (80%) said they would be very likely to use the test again if their doctor ordered it.
Schroy & Heeren (2005) [21]	Asymptomatic patients 50 years of age and older at mostly average risk for CRC (n = 4042)	Prospective survey	Patients completed a survey after undergoing sDNA, fecal occult blood test (FOBT), and colonoscopy	45% of patients preferred sDNA testing, 32% preferred FOBT, and 15% preferred colonoscopy. 8% had no preference. sDNA testing had higher ratings for prep and test related features. Colonoscopy was rated higher for perceived accuracy. Preference for sDNA testing did not differ significantly across age, gender, or race/ethnicity.
Schroy et al., (2007) [22]	Asymptomatic patients between the ages of 50 and 75 who had no prior screening for CRC other than FOBT (n = 263)	Cross-sectional survey	Patients reviewed a decision aid with a research assistant and were then asked to complete a survey to indicate their preferred CRC screening test and reasons for their choice	50.6% of patients preferred colonoscopy, citing accuracy. 28.1% and 18.3% preferred sDNA and FOBT, respectively, citing concerns about discomfort and frequency of testing.
Yang et al., (2014) [23]	1200 randomly selected patients stratified equally by gender and by the following age groups: 50–59, 60–69, and 70–79 received surveys. Only those returned were included. (n = 434)	Cross-sectional survey	Patients completed a survey regarding knowledge of, personal and family history of, and personal concern for cancer as well as past CRC screening behavior and interest in multi-organ stool DNA test (MUST)	98% of patients said they would use MUST. Reasons, in order of importance, were: multi-cancer detection, absence of bowel preparation, safety and noninvasiveness. MUST was preferred over colorectal-only sDNA testing (53%), occult blood testing (75%), colonoscopy (84%), sigmoidoscopy (91%), and barium enema (95%). 9% of respondents indicated that fear of finding cancer was a concern with MUST, and only 3% indicated unpleasantness of stool sampling as a potential barrier.

## Data Availability

Not applicable.
